# Scientific Value of Systematic Reviews: Survey of Editors of Core Clinical Journals

**DOI:** 10.1371/journal.pone.0035732

**Published:** 2012-05-01

**Authors:** Joerg J. Meerpohl, Florian Herrle, Gerd Antes, Erik von Elm

**Affiliations:** 1 German Cochrane Center, Institute of Medical Biometry and Medical Informatics, University Medical Center Freiburg, Freiburg, Germany; 2 Pediatric Hematology and Oncology, Center for Pediatrics and Adolescent Medicine, University Medical Center Freiburg, Freiburg, Germany; 3 Department of Surgery, University Medical Center Mannheim, University of Heidelberg, Mannheim, Germany; 4 Cochrane Switzerland, IUMSP, University Hospital Lausanne, Lausanne, Switzerland; McGill University Health Centre, McGill University, Canada

## Abstract

**Background:**

Synthesizing research evidence using systematic and rigorous methods has become a key feature of evidence-based medicine and knowledge translation. Systematic reviews (SRs) may or may not include a meta-analysis depending on the suitability of available data. They are often being criticised as ‘secondary research’ and denied the status of original research. Scientific journals play an important role in the publication process. How they appraise a given type of research influences the status of that research in the scientific community. We investigated the attitudes of editors of core clinical journals towards SRs and their value for publication.

**Methods:**

We identified the 118 journals labelled as “core clinical journals” by the National Library of Medicine, USA in April 2009. The journals’ editors were surveyed by email in 2009 and asked whether they considered SRs as original research projects; whether they published SRs; and for which section of the journal they would consider a SR manuscript.

**Results:**

The editors of 65 journals (55%) responded. Most respondents considered SRs to be original research (71%) and almost all journals (93%) published SRs. Several editors regarded the use of Cochrane methodology or a meta-analysis as quality criteria; for some respondents these criteria were premises for the consideration of SRs as original research. Journals placed SRs in various sections such as “Review” or “Feature article”. Characterization of non-responding journals showed that about two thirds do publish systematic reviews.

**Discussion:**

Currently, the editors of most core clinical journals consider SRs original research. Our findings are limited by a non-responder rate of 45%. Individual comments suggest that this is a grey area and attitudes differ widely. A debate about the definition of ‘original research’ in the context of SRs is warranted.

## Introduction

Since the first comparative study to answer a therapeutic question by James Lind in the 18^th^ century [1], the number of medical research studies is ever increasing. Accordingly, the need to synthesize research evidence has been recognized for over two centuries. A more formal approach to systematically synthesizing the research evidence that accumulates in medical science in a systematic review was not developed until the late 20^th^ century and gained momentum with the advent of evidence-based medicine. [2] Besides providing a comprehensive overview, systematic reviews help to identify areas where further research is needed or, inversely, might be unnecessary or even unethical [3].

Evidence-based medicine has been called a “new paradigm” because it asks questions about health care in an answerable format and considers the best evidence available from clinical research. In order to keep abreast of the large quantity of new data being generated continuously, systematic reviews have become the central and indispensable tool of evidence-based medicine [4].

In contrast to classical narrative reviews, systematic reviews use an explicit and rigorous methodology. They start with a clearly stated set of clinically relevant questions and pre-defined criteria for study inclusion. The scientific literature is then systematically searched with the aim of identifying all potentially relevant studies. After application of eligibility criteria, the included studies are assessed for their internal validity, in particular the risk of bias. If possible, data are combined using meta-analytic methods. [5] By statistically combining information from all or part of the included studies, meta-analyses can provide pooled estimates that are more precise than those derived from individual studies. [6] Presence or absence of a meta-analysis does not represent a quality criterion since it is directly dependent on the studies identified and data available for inclusion in the systematic review. Finally, results of any systematic review need to be interpreted in the light of random error and considering external validity or applicability of results.

The general concept of systematic reviews and their methodology were originally developed in the social sciences. In medicine they have been applied predominantly for the evaluation of treatments. [5] With some adaptations the general methodology of systematic reviews is also applicable to questions of diagnostic test accuracy and prognosis. [7]–[9] Systematic reviews usually provide more reliable findings than individual studies or non-systematic narrative reviews. [10]–[13] Consequently, more robust conclusions can be drawn which, in turn, may inform decision making on different levels from individual patient care to the organization of health care systems.

Over the last 10 to 15 years, the number of published systematic reviews has increased markedly. [14] When carried out before the start of new clinical studies, a systematic review can help to optimize the allocation of limited research resources. Consequently, leading funding agencies such as the UK Medical Research Council require systematic reviews as part of grant applications. [15] Leading medical journals now advocate a systematic overview of the evidence as part of published reports of new randomized trials. [16] In recent years the role of research syntheses has been further strengthened by the decision of the U.S. government in 2009 to allocate $1.1 billion to comparative-effectiveness research (CER) under the framework of the American Recovery and Reinvestment Act. [17] The Institute of Medicine (IOM) recently published two reports which underline the relevance of systematic review methodology both for CER and evidence-based clinical practice guidelines [18], [19].

Despite these important functions and the recent prominent government support, the status and value of systematic reviews is still being disputed in academia. The debate is partly fueled by persistent misconceptions. [20] In the past, systematic reviews have been dubbed “secondary research” in contrast to “primary or original research”, implying that they were less scientifically novel and required less methodological rigor than studies deemed primary research. Early opponents even spoke of “mega-silliness” and “statistical alchemy for the 21^st^ century” [21].

At present there is considerable heterogeneity across countries with regard to both the funding available for systematic reviews and their academic recognition. While in some countries, such as the U.K. and The Netherlands, medical faculties have established professorships and academic units for systematic reviews the related methods still play only a minor role in medical student education in other countries. Similarly, the way methods of research synthesis are adopted and used varies widely across medical specialty fields.

Scientific journals play an important role in the dissemination of scientific knowledge by setting quality standards and determining the way in which research is being published. Journal editors can thus be considered gatekeepers. How they appraise a given type of research activity influences the recognition it receives in the scientific community. Given the influential role of journal editors, we set out to elucidate their attitudes towards systematic reviews. In particular, we were interested in the scientific status attributed to systematic reviews and their acceptability for publication in clinical journals.

## Methods

We identified the 118 core clinical journals as defined by the National Library of Medicine (USA) as of April 2009 (see File S1). From the journals’ websites we retrieved the contact details of the main editorial offices and, if available, of the editors-in-chief. We sent out a short email questionnaire in April 2009 asking whether editors would

1. consider a systematic review manuscript an original research project
2. publish a systematic review in their journal, and
3. in which section of their journal they would publish a systematic review.

A reminder email was sent in August 2009. For all but two journals, the editors-in-chief responded to our survey; in the remaining two journals other editorial staff answered the survey. Ethics approval was not required since editors-in-chief were free to participate in our survey and data were anonymized.

Two investigators then independently evaluated and classified the responses. If discrepancies occurred, consensus was reached in discussion with a third investigator. We extracted the ISI impact factor of the included journals from the Journal Citation Report 2009. [22] All data were collated in a spreadsheet in Microsoft Excel and used for descriptive statistics.

To characterize the group of non-responding journals, we developed a three step process that entailed 1) a PubMed search for systematic reviews classified as meta-analyses published in these journals in 2009, 2) hand-searching the content published in 2009 of journals for which we did not identify a meta-analysis in our PubMed search, 3) evaluation of author instructions of journals from point 2 (above) to determine whether they would have published systematic reviews.

## Results

Seventeen (14%) of the 118 journals were general medical journals and 101 (86%) were specialty journals. The majority of the journals were published in the USA (Table 1). Editors of 65 journals (55%) responded to our survey. For three journals, not all questions were answered. The response rate was higher for editors of general medical journals (13/17; 76%) than for those of specialty journals (52/101; 52%). We received responses from 50% (51/101) of the U.S. journals and from 80% (12/15) of the British journals. The median ISI impact factor for responder journals was 2.99 (range 0.29 – 52.6) and for non-responder journals 3.61 (range 0.40 – 69.0).

**Table 1:** Journal characteristics by survey responder status.

JOURNAL CHARACTERISTICS	RESPONDERS	NON-RESPONDERS	TOTAL
	N = 65 (55%)	N = 53 (45%)	N = 118
**Type of journal**			
General medical journal*	13 (76%)	4 (24%)	17
(Sub-) Speciality journal**	52 (52%)	49 (48%)	101
**Country of publication**			
USA	51 (50%)	50 (50%)	101
United Kingdom	12 (80%)	3 (20%)	15
Other	2 (100%)	0 (0%)	2
**Journal impact factor**			
JCR 2007 - median (range)	2.99 (0.29–52.59)	3.60 (0.37–69.03)	3.23 (0.29–69.03)

* e.g. JAMA, BMJ, NEJM;

** e.g., Annals of Surgery, Chest, Rheumatology.

### Status of Systematic Reviews

Seventy-one percent (46/65) of editors regarded systematic reviews as original research projects. Nine of them (29%) did so only under certain premises (Table 2). For some editors the use of Cochrane methodology [5] or meta-analytic methods were a criterion to decide whether a systematic review is considered original research. For illustration, Table 3 includes some excerpts from the responses.

**Table 2:** Status of systematic reviews – results of survey of journal editors.

SURVEY QUESTION	ANSWERS N = 65
1. “Do you consider a systematic review an original research project?”	Yes: 37 (57%)
	Under premises: 9 (14%)
	No: 19 (29%)
2. “Do you publish systematic reviews in your journal?”	Yes: 54 (83%)
	Rarely: 6 (9%)
	No: 4 (6%)
	Answer missing: 1 (2%)
3. “For which section would you consider a manuscript reportingon a systematic review?”*	(Systematic) review: 23 (35%)
	(Original) research: 18 (28%)
	Special article: 9 (14%)
	By topic: 9 (14%)
	Does not apply: 5 (8%)
	Answer missing: 3 (5%)

* multiple answers possible.

**Table 3:** Comments illustrating editors’ attitudes towards systematic reviews.

Editor considered systematic reviews original research	Editor did not consider systematic reviews original research
“Systematic reviews such as a Cochrane meta-analysis is an original research article.”	“By definition, a “review” is not original research. If a systematic review yields new knowledge, it may be considered research in some fields of inquiry”
“… we do consider complex systematic reviews with protocols and the like research projects.”	“…we are interested in original observations and new mechanisms of disease.”
“I consider them important scholarly work.”	“We agree that a systematic review is rigorous scholarship and that a narrative review can be. But that is not the same as the “scholarship of discovery”
“Grey area, but we consider this original”	“We are unsettled in our opinion. To some extent it depends on the manner in which the review was conducted: a strictly statistical analysis shows little intellectual input, is seldom of value and always says ‘more research is needed’.”
“…we DO consider meta-analyses as original research. However, a systematic review without a meta-analysis is considered a Review Article”	–

### Acceptability for Publication

Of 64 respondents, 60 (94%) published systematic reviews (Table 2). Six (9%) did so only rarely and four (6%) not at all. About a third of the journals published systematic reviews in a section dedicated to original research articles and a third in a specific section for (systematic) reviews. Some journals either featured them as special articles or placed them in other sections (Table 2).

### Non-responding Journals

Of the 53 journals that did not respond to our survey, 30 (56.6%) published at least one systematic review in 2009, two (3.8%) would accept systematic reviews according to their author instructions, while 21 (39.6%) neither published any nor explicitly mention systematic reviews in their author instructions. Of those that published systematic reviews, in 18 of the 30 the systematic reviews were published in journal sections that contained original research articles.

## Discussion

We surveyed editors of core clinical journals and found that most of them regarded systematic reviews as original research. Nearly all of the journals represented by these editors published systematic reviews. The respective comments of the respondents indicate that this is an ill-defined area. This was mirrored by the variety of criteria used to decide whether a submitted systematic review manuscript represented original research or not. For some respondents, the inclusion of a meta-analysis was the key argument while others looked at the methods being used.

In our set of core clinical journals the general attitude towards systematic reviews was rather positive. It is conceivable that a broader sample of biomedical journals, e.g., including basic sciences journals, would have yielded a more conservative picture. The main limitation of our survey is that about 45% of the contacted journals did not respond which could potentially significantly change the results and affect the interpretation. While the proportion of non-responding journals that published systematic reviews was lower, the majority still published at least one in 2009. Interestingly, more than half of the non-responder journals that published systematic reviews seemed to consider them original research. If one assumes that the non-responding editors are more skeptical about systematic reviews than those who responded then the results may be less positive overall.

From the large spectrum of responses we conclude that a debate about the status and the academic recognition of systematic reviews is warranted. A next step should be an in-depth analysis of the views of different stakeholders including researchers, funders, users of systematic reviews (e.g., policy makers) and again journal editors.

Ideally, the clinical research community would accept systematic reviews as a research category of its own, which is defined by methodological criteria, as is the case for other types of research. With certain quality criteria being fulfilled, systematic reviews should not be denied the appropriate academic recognition they deserve. Under these premises their scientific value should be on par with conventional original research studies. This argument becomes even more compelling when one considers that systematic reviews are essentially observational studies of aggregate or individual data from previous studies.

Due to the continuous work of The Cochrane Collaboration and other international institutions and networks, the use and recognition of systematic reviews has increased considerably over the last 15 years. [4] However, the limited funding opportunities available for systematic review projects represent a main barrier to an even wider implementation. A clarification of the scientific status of systematic reviews might motivate researchers to undertake such projects to an even larger extent. If high-quality systematic reviews are accepted as valid research projects by the research community, then funding agencies might also be more open to financially support them e.g. by creating specific grant schemes.

In conclusion, the attitudes of editors of clinical journals vary with regard to the value given to systematic reviews. Most responding editors regarded systematic reviews as original research projects based on varying criteria. This interpretation is limited by a non-responder rate of 45%. A debate about the scientific value of systematic reviews and their academic recognition is warranted and would help establish sustainable programs of evidence synthesis across countries and different fields of clinical research.

## Supporting Information

File S1 Journals invited to participate in survey.
(DOCX)
