# Correction: Scientific Value of Systematic Reviews: Survey of Editors of Core Clinical Journals

**DOI:** 10.1371/annotation/b9a9cb87-3d96-47e4-a073-a7e97a19f47c

**Published:** 2012-10-04

**Authors:** Joerg J. Meerpohl, Florian Herrle, Stefan Reinders, Gerd Antes, Erik von Elm

Author Stefan Reinders was erroneously omitted from the published author list. The correct byline is: Joerg J. Meerpohl, Florian Herrle, Stefan Reinders, Gerd Antes, Erik von Elm. Stefan Reinders' affiliation is: London School of Hygiene and Tropical Medicine, London, United Kingdom.

The correct affiliations list is:

1 German Cochrane Center, Institute of Medical Biometry and Medical Informatics, University Medical Center Freiburg, Freiburg, Germany,

2 Pediatric Hematology and Oncology, Center for Pediatrics and Adolescent Medicine, University Medical Center Freiburg, Freiburg, Germany,

3 Department of Surgery, University Medical Center Mannheim, University of Heidelberg, Mannheim, Germany,

4 London School of Hygiene and Tropical Medicine, London, United Kingdom,

5 Cochrane Switzerland, IUMSP, University Hospital Lausanne, Lausanne, Switzerland

The correct citation is: Meerpohl JJ, Herrle F, Reinders S, Antes G, von Elm E (2012) Scientific Value of Systematic Reviews: Survey of Editors of Core Clinical Journals. PLoS ONE 7(5): e35732. doi:10.1371/journal.pone.0035732

Stefan Reinders' contributions are: Performed the experiments, analyzed the data, and Other: Contributed substantially to collection of information (journal characteristics) on 118 core clinical journals and preparation/conduct of survey, Involved in collection/collation of survey results, Involved in descriptive analysis of data, Involved in writing of the manuscript and approved final manuscript. 

