# Supplementary material for: Scientific Value of Systematic Reviews: Survey of Editors of Core Clinical Journals
Source: PLoS One. 2012 May 1;7(5):e35732. doi: 10.1371/journal.pone.0035732 (PMC3341385; doi:10.1371/journal.pone.0035732)
Supplement: File S1 — Journals invited to participate in survey. (DOCX) [file pone.0035732.s001.docx]

| **#** | **Journal Title (NLM-Abbreviation)** |
| --- | --- |
| 1 | Acad Med |
| 2 | Am Fam Physician |
| 3 | Am Heart J |
| 3 | Am J Cardiol |
| 4 | Am J Clin Nutr |
| 5 | Am J Clin Pathol |
| 6 | Am J Med |
| 7 | Am J Med Sci |
| 8 | Am J Nurs |
| 9 | Am J Obstet Gynecol |
| 10 | Am J Ophthalmol |
| 11 | Am J Pathol |
| 12 | Am J Phys Med Rehabil |
| 13 | Am J Psychiatry |
| 14 | Am J Public Health |
| 15 | Am J Respir Crit Care Med |
| 16 | Am J Roentgenol |
| 17 | Am J Surg |
| 18 | Am J Trop Med Hyg |
| 19 | Anaesthesia |
| 20 | Anesth Analg |
| 21 | Anesthesiology |
| 22 | Ann Emerg Med |
| 23 | Ann Intern Med |
| 24 | Ann Oto Rhinol Laryn |
| 25 | Ann Surg |
| 26 | Ann Thorac Surg |
| 27 | Arch Dermatol |
| 28 | Arch Dis Child |
| 29 | Arch Dis Child-Fetal |
| 30 | Arch Environ Occup H |
| 31 | Arch Gen Psychiatry |
| 32 | Arch Intern Med |
| 33 | Arch Neurol-Chicago |
| 34 | Arch Ophthalmol |
| 35 | Arch Otolaryngol Head Neck Surg |
| 36 | Arch Pathol Lab Med |
| 37 | Arch Pediatr Adolesc Med |
| 38 | Arch Phys Med Rehabil |
| 39 | Arch Surg |
| 40 | Arthritis Rheum |
| 41 | BJOG |
| 42 | Blood |
| 43 | BMJ |
| 44 | Br J Surg |
| 45 | Brain |
| 46 | CA Cancer J Clin |
| 47 | Cancer |
| 48 | Chest |
| 49 | Circulation |
| 50 | Clin Orthop Relat Res |
| 51 | Clin Pediatr (Phila) |
| 52 | Clin Pharmacol Ther |
| 53 | Clin Toxicol (Phila) |
| 54 | CMAJ |
| 55 | Crit Care Med |
| 56 | Curr Probl Surg |
| 57 | Diabetes |
| 58 | Dig Dis Sci |
| 59 | Dis Mon |
| 60 | Endocrinology |
| 61 | Gastroenterology |
| 62 | Geriatrics |
| 63 | Gut |
| 64 | Heart |
| 65 | Heart Lung |
| 67 | Hosp Health Netw |
| 68 | J Allergy Clin Immunol |
| 69 | J Am Coll Cardiol |
| 70 | J Am Coll Surg |
| 71 | J Am Diet Assoc |
| 72 | J Bone Joint Surg Am |
| 73 | J Bone Joint Surg Br |
| 74 | J Clin Endocrinol Metab |
| 75 | J Clin Invest |
| 76 | J Clin Pathol |
| 77 | J Fam Pract |
| 78 | J Gerontol A Biol Sci Med Sci |
| 79 | J Gerontol B Psychol Sci Soc Sci |
| 80 | J Immunol |
| 81 | J Infect Dis |
| 82 | J Laryngol Otol |
| 83 | J Nerv Ment Dis |
| 84 | J Neurosurg |
| 85 | J Nurs Adm |
| 86 | J Oral Maxillofac Surg |
| 87 | J Pediatr |
| 88 | J Thorac Cardiovasc Surg |
| 89 | J Trauma |
| 90 | J Urol |
| 91 | JAMA |
| 92 | Lancet |
| 93 | Mayo Clin Proc |
| 94 | Med Clin North Am |
| 95 | Med Lett Drugs Ther |
| 96 | Medicine (Baltimore) |
| 97 | N Engl J Med |
| 98 | Neurology |
| 99 | Nurs Clin North Am |
| 100 | Nurs Outlook |
| 101 | Nurs Res |
| 102 | Obstet Gynecol |
| 103 | Orthop Clin North Am |
| 104 | Pediatr Clin North Am |
| 105 | Pediatrics |
| 106 | Phys Ther |
| 107 | Plast Reconstr Surg |
| 108 | Postgrad Med |
| 109 | Prog Cardiovasc Dis |
| 110 | Public Health Rep |
| 111 | Radiol Clin North Am |
| 112 | Radiology |
| 113 | Rheumatology (Oxford) |
| 114 | South Med J |
| 115 | Surg Clin North Am |
| 116 | Surgery |
| 117 | Transl Res |
| 118 | Urol Clin North Am |
